# Construction of circRNA-miRNA-mRNA Network for Exploring Underlying Mechanisms of Lubrication Disorder

**DOI:** 10.3389/fcell.2021.580834

**Published:** 2021-03-11

**Authors:** Shengnan Cong, Jinlong Li, Jingjing Zhang, Jingyi Feng, Aixia Zhang, Lianjun Pan, Jiehua Ma

**Affiliations:** ^1^School of Nursing, Nanjing Medical University, Jiangsu, China; ^2^Department of Laboratory Medicine, The Second Hospital of Nanjing, Nanjing University of Chinese Medicine, Nanjing, China; ^3^Women’s Hospital of Nanjing Medical University (Nanjing Maternity and Child Health Care Hospital), Nanjing, China; ^4^High School Affiliated To Nanjing Normal University International Department, Nanjing, China

**Keywords:** lubrication disorder, non-coding RNA, circular RNA, microRNA, competing endogenous RNA

## Abstract

Lubrication disorder is a common health issue that manifests as insufficient sexual arousal at the beginning of sex. It often causes physical and psychological distress. However, there are few studies on lubrication disorder, and the complexity of circular RNA (circRNA) and the related competing endogenous RNA (ceRNA) network in lubrication disorder is still poorly known. Therefore, this study aims to build a regulatory circRNA-micro (mi)RNA-mRNA network and explore potential molecular markers of lubrication disorder. In the study, 12 subjects were recruited, including 6 in the lubrication disorder group and 6 in the normal control group. RNA sequencing was exploited to identify the expression profiles of circRNA, miRNA and mRNA between two groups, and then to construct the circRNA-miRNA-mRNA networks. The enrichment analyses of the differentially expressed (DE)-mRNAs were examined via Gene Set Enrichment Analysis (GSEA). Furthermore, the expression level and interactions among circRNA, miRNA, and mRNA were validated using real-time quantitative polymerase chain reaction (RT-qPCR) and dual-luciferase reporter assays. In the results, 73 circRNAs, 287 miRNAs, and 354 target mRNAs were differentially expressed between two groups when taking | Log2 (fold change)| > 1 and *P*-value < 0.05 as criteria, and then the results of GSEA revealed that DE-mRNAs were linked with “vascular smooth muscle contraction,” “aldosterone regulated sodium reabsorption,” “calcium signaling pathway,” etc. 19 target relationships among 5 circRNAs, 4 miRNAs, and 7 mRNAs were found and constructed the ceRNA network. Among them, hsa-miR-212-5p and hsa-miR-874-3p were demonstrated to be related to the occurrence of lubrication disorder. Eventually, consistent with sequencing, RT-qPCR showed that hsa_circ_0026782 and *ASB2* were upregulated while hsa-miR-874-3p was downregulated, and dual-luciferase reporter assays confirmed the interactions among them. In summary, the findings indicate that the circRNA-miRNA-mRNA regulatory network is presented in lubrication disorder, and ulteriorly provide a deeper understanding of the specific regulatory mechanism of lubrication disorder from the perspective of the circRNA-miRNA-mRNA network.

## Introduction

Female sexual dysfunction (FSD) is regarded as a common health problem, which results from the interplay of multiple factors, such as age, race, menstrual status, relationships, mental health, etc. (Ma et al., 2015, 2014). It affects conjugal sexual relationships, which leads to interpersonal difficulties and causes obvious pain to patients (Miller et al., 2018; Clayton and Valladares Juarez, 2019). In the fifth edition of the Diagnostic and Statistical Manual of Mental Disorders (DSM-5), FSD was classified as three aspects: Female Sexual Interest-Arousal Disorder, Female Orgasmic Disorder and Genito-Pelvic Pain-Penetration Disorder (McCabe et al., 2016). At present, the incidence of FSD is temporarily uncertain, and the reports of it vary widely, with estimates ranging from 25.8% to 91.0% (Clayton and Valladares Juarez, 2019). However, FSD is not taken seriously in China. Under the influence of traditional Chinese culture, women are ashamed to express their troubles and appeals, which makes it difficult to diagnose and treat diseases, and makes women with sexual dysfunction not get the proper help and solutions in sexual problems (Zhang J. et al., 2019). In a previous cross-sectional study of the Chinese population, boundary value scores for the Chinese Version of the FSFI (CVFSFI) were established. Based on this standard, the prevalence of lubrication disorder, orgasmic disorder, arousal disorder, low interest and sexual pain among urban Chinese women were 36.8, 30.6, 25.4, 23.6, and 21.8%, respectively (Ma et al., 2014). Lubrication disorder (LD), which is mainly manifested as the failure to obtain or maintain vaginal wetness in sexual activity (Graham, 2010), has the highest prevalence, so it should be paid more attention to. However, the specific pathogenesis needs to be further elucidated to understand the occurrence of LD.

In the last few years, with the emergence of advanced gene analysis techniques, non-coding RNA has been found to play an essential role in human diseases as a gene regulator (Mattick and Makunin, 2006; Bartoszewski and Sikorski, 2018), especially circular RNA (circRNA) and microRNA (miRNA) have attracted great attention (Bernardo et al., 2015; Beermann et al., 2016; Mishra et al., 2016; Cao and Zhen, 2018; Chen and Huang, 2018). CircRNA is a circular non-coding RNA, and it is highly abundant and stable in organisms by reason of its covalently closed-loop structure (Meng et al., 2017; Kristensen et al., 2019; Zhang J. et al., 2019). Recently, circRNA has been the research hotspot, and our previous study has also demonstrated the existence of differentially expressed circRNAs in the vaginal epithelium of women with LD, but only verified their expression level (Zhang J. et al., 2019). MiRNA, a small non-coding RNA, is composed mainly of 19-25 nucleotides and plays an important role in cell proliferation, differentiation, apoptosis, and maintenance of immune homeostasis (Chen et al., 2016). Unfortunately, no one has yet explored the function of miRNAs in the field of LD, except for one of our previous works. In our previous research, we have validated that the overexpression of miR-137 could reduce cell permeability, and Aquaporin-2 (*AQP2*) was considered to be a target of miR-137 (Zhang H. et al., 2018), which suggested that miRNA may be involved in the occurrence and development of LD by down-regulating protein levels. All of the above results indicate that circRNAs and miRNAs may be related to the pathogenesis of LD, and simultaneously provide an idea for us to prove the interactions between them.

Currently, the competing endogenous RNA (ceRNA) hypothesis has revealed a new mechanism for RNA interactions in many diseases (Tay et al., 2014; Jiang and Yuan, 2019). CeRNA is not specifically RNA (Tay et al., 2014; An et al., 2017). It can competitively combine with miRNAs through microRNA response elements (MREs), thereby inhibiting gene silencing by isolating miRNAs from messenger RNAs (mRNAs)(Karreth and Pandolfi, 2013; Tay et al., 2014; An et al., 2017). Likewise, multiple studies have also confirmed that circRNA can function as ceRNA in many diseases because it can regulate the expressions of mRNAs by acting as sponges for miRNAs (Hansen et al., 2013; Han et al., 2018; Panda, 2018; Zhong et al., 2018). For example, Zhang M. et al. (2018) revealed that circRNA_103237 can inhibit cell proliferation, migration and cell cycle maintenance in early secretory endometrium of women with endometriosis through absorbing miR-34 (Burney et al., 2009). The evidence suggests that such a circRNA-miRNA-mRNA competing system plays an important role in disease (Hu et al., 2018; Zhang Y. et al., 2019). However, the complexity of circRNA and the related ceRNA network in lubrication disorder is still poorly known.

**FIGURE 1:**
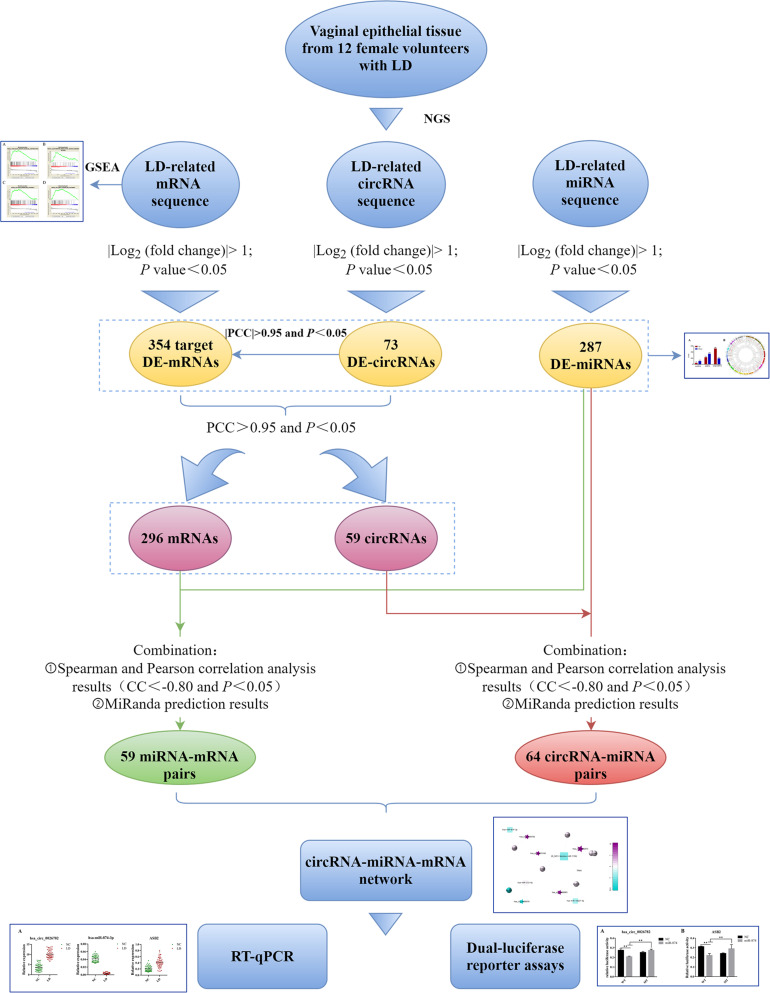
Flow chart of this study. LD, lubrication disorder; NGS, next-generation sequencing; circRNA, circular RNA; miRNA, microRNA; mRNA, messenger RNA; DE, differentially expressed; GSEA, Gene Set Enrichment Analysis; PCC, Pearson correlation coefficient; CC, correlation coefficient; RT-qPCR, real-time quantitative polymerase chain reaction.

Therefore, in order to explore the ceRNA network in LD and further understand the potential mechanism of LD development, we identified differentially expressed miRNAs, circRNAs and mRNAs in the vaginal epithelium of women between the LD group and normal control group. Thereafter, we conducted Gene Set Enrichment Analysis (GSEA) on differentially expressed mRNAs to describe their biological functions. According to the ceRNA hypothesis, the circRNA-miRNA-mRNA network was established. Finally, real-time quantitative polymerase chain reaction (qRT-PCR) and dual-luciferase reporter assays were implemented to verify the accuracy of the network (Figure 1).

## Materials and Methods

### Sample Source

Vaginal epithelial tissue from 12 female volunteers was collected at Nanjing Maternity and Child Health Care Hospital (AKA: Women’s Hospital of Nanjing Medical University). The inclusion criteria for women are as follows: (1) Adult Chinese women from Han nationality with secondary education or above; (2) without any other gynecological diseases; (3) planned to undergo vaginal tightening surgery. Women included in the study should be evaluated using the Chinese version of the Female Sexual Function Index scale (CVFSFI). Then in line with the cutoff point of 4.05 reported by Ma et al. (2014), the subjects with scale scores lower than 4.05 were assigned to the LD group, while the opposites were divided into the normal control group. Finally, each group had 6 participants and their basic characteristics were presented in Table 1. Vaginal epithelial tissue from two groups was collected by a professional surgeon and then kept in liquid nitrogen immediately during the operation. Once the operation is over, the tissue was stored at −80°C. The experiment was licensed by the Ethics Review Committee of Nanjing Maternity and Child Health Care Hospital.

**TABLE 1:** Basic characteristics and FSFI scale scores of subjects in lubrication disorder and normal control group.

**Group**	**Average age**	**Lubrication**	**Desire**	**Arousal**	**Orgasm**	**Sexual pain**	**Satisfaction**
LD	45	3.35 ± 0.48**	2.50 ± 0.80	2.95 ± 0.84	3.73 ± 0.33	3.73 ± 0.97**	4.20 ± 0.55
NC	46	5.10 ± 0.50	3.40 ± 1.05	3.30 ± 0.76	4.27 ± 1.35	5.67 ± 0.39	5.13 ± 0.93

FSFI, female sexual function index; LD, lubrication disorder; NC, normal control.

**P-value < 0.01.

### RNA Extraction and Next-Generation Sequencing (NGS)

Total sample RNAs were obtained using QIAzol Lysis Reagent (Qiagen, Germantown, MD, United States) as instructed by the manufacturer. The quality and quantity of total RNAs were detected through a Nanodrop ND-1000 spectrophotometer (Thermo Fisher Scientific, Carlsbad, CA, United States), whereas the integrity of RNAs was verified with standard denaturing 1% agarose gel electrophoresis, Agilent 2100 BioAnalyzer (Agilent Technologies, Santa Clara, CA, United States) and Qubit Fluorometer (Invitrogen). Only when the total RNA samples meet the requirements of RNA integrity number (RIN) > 7.0 and a 28S:18S ratio > 1.8, they would be applied to the subsequent experiments.

After RNA extraction, constructions of cDNA libraries and next-generation sequencing were all performed by CapitalBio Technology (Beijing, China). Briefly, for circRNA and mRNA, ribosomal RNAs in total RNAs were removed by Ribo-Zero^TM^ Magnetic Kit (Epicenter), and then the remaining RNA was cut randomly into short fragments. M-MuLV Reverse Transcriptase (RNaseH-) and random hexamer primer were exploited to synthesize the first-strand cDNA. Subsequently, DNA Polymerase I and RNase H were used for performing the second-strand cDNA synthesis. dNTPs with dTTP were replaced by dUTP in the reaction buffer. Then, via exonuclease/polymerase activities, the remaining overhangs were converted into blunt ends. NEBNext Adaptor with hairpin loop structure was ligated to prepare for hybridization after adenylation of 3′ ends of DNA fragments. In order to screen cDNA fragments of 150–200 bp in length, the library fragments were purified using the AMPure XP system (Beckman Coulter, Beverly, United States). Whereafter, 3 μl USER Enzyme (NEB, United States) was used with size-selected, adaptor-ligated cDNA at 37°C for 15 min followed by 5 min at 95°C before PCR. Phusion High-Fidelity DNA polymerase, Index (X) Primer, and Universal PCR primers were utilized to perform PCR. Finally, we purified the products (AMPure XP system) and assessed the library quality on the Agilent Bioanalyzer 2100 system.

For miRNA, NEB 3′ SR Adaptor was directly and specifically ligated to 3′ end of miRNA, siRNA and piRNA. Following the 3′ ligation reaction, the excess of 3′ SR Adaptor (that remained free after the 3′ ligation reaction) was explored to hybridize the SR RT Primer, and then the single-stranded DNA adaptor was transformed into a double-stranded DNA molecule. 5′ends adapter was ligated to 5′ends of miRNAs, siRNA, and piRNA. Afterward, using M-MuLV Reverse Transcriptase (RNase H−), the first-strand cDNA was synthesized. Long Amp Taq 2X Master Mix, SR Primer for illumina, and index (X) primer were used for performing PCR amplification. And the products were purified through an 8% polyacrylamide gel (100V, 80 min). 140∼160 bp (the length of small non-coding RNA plus the 3′ and 5′ adaptors) DNA fragments were obtained and dissolved in 8 μL elution buffer. Ultimately, the Agilent Bioanalyzer 2100 system was used to assess the library quality using DNA High Sensitivity Chips.

### Differentially Expression Analysis

The expressions of miRNAs, circRNAs and mRNAs in the LD group was compared with that in the normal control group. R software with edgeR/limma packages was utilized to perform differential expression analysis, and the criteria of | Log_2_ (fold change)| > 1 and *P*-value < 0.05 were adopted to measure significantly differentially expressed (DE)-circRNAs, DE-miRNAs and DE-mRNAs between two groups. At the same time, target DE-mRNAs were also screened by correlation analysis between circRNA and mRNA with the criteria of | PCC| > 0.95 and *P* < 0.05. Finally, circos plot which presented the differential expression and distribution of circRNAs and target mRNAs was rendered by Circos software.

### Gene Set Enrichment Analysis of DE-mRNA

Gene Set Enrichment Analysis^1^ was utilized to analyze the potential pathways of DE-mRNAs in LD. The data was normalized 1000 times to get the normalized enrichment score (NES) according to the default weighted enrichment statistical method. When | NES| >1, the pathway were statistically significant and the gene sets would be selected.

### Co-expression Analysis of DE-circRNAs and DE-mRNAs

Co-expression analysis was implemented by calculating the Pearson correlation coefficient (PCC) based on the expression levels of DE-circRNAs and target DE-mRNAs. The t.test function in the R data analysis tool was used to evaluate. Subsequently, with the standard of PCC > 0.95 and *P* < 0.05, we would retain the positively related DE-circRNAs and DE-mRNAs for further study.

### Construction of circRNA-miRNA Pairs

We constructed pairs by selecting circRNAs that were differentially expressed and positively correlated with mRNAs. To discover such circRNA-miRNA interactions, we applied both Pearson and Spearman, simultaneously. By using the function t.test in the R data analysis tool, we computed the correlation coefficient between DE-circRNAs and DE-miRNAs according to their sequencing results and screened out negatively correlated pairs with both defaults filtering criteria of correlation coefficient (CC) < −0.80 and *P* < 0.05. MiRanda software was used to predict the target miRNAs of circRNAs (Li X. N. et al., 2018; Xiu et al., 2019). Finally, by taking the intersection with the results of the previous steps, highly correlated circRNA-miRNA pairs were built.

### Construction of miRNA-mRNA Pairs

Similarly, by joint differentially expressed mRNAs data and co-expression mRNAs data, mRNAs were chosen and both Pearson and Spearman correlation analyses were used to determine the relationships between DE-miRNAs and the selected mRNAs. The function t.test in the R data analysis tool was exploited to calculate CC, and pairs with significant negative correlation were filtered according to CC < −0.80 and *P* < 0.05. Meanwhile, the target mRNAs of miRNAs were predicted by MiRanda software. Eventually, by merging the above two results, miRNA-mRNA pairs were established.

### Construction of the ceRNA Network

The ceRNA regulatory network was built by merging the pairs of miRNA-circRNA and miRNA-mRNA. The nodes that didn’t overlap would be removed. Cytoscape software was used to visualize the network.

### Real-Time Quantitative Polymerase Chain Reaction (RT-qPCR)

A total of 32 samples are used for verification using RT-qPCR. Total RNAs from vaginal epithelial tissue were extracted using RNA-easy^TM^ isolation reagent R701 (Vazyme Biotech, Nanjing, China) in line with the manufacturer’s protocol. Then they were applied to synthesize the cDNA with RT-PCR Kit (Vazyme Biotech, Nanjing, China). For miRNA analysis, reverse transcription steps of cDNA were performed according to the manufacturer’s instructions (Vazyme Biotech, Nanjing, China). And HiScript^®^ III RT SuperMix for qPCR (+ gDNA wiper) (Vazyme Biotech, Nanjing, China) were used for circRNA and mRNA analysis. The divergent primers were design and synthesis by Realgene (Nanjing, China), and the sequences of the primers are shown in Table 2. qPCR was conducted on Applied Biosystems ViiA system (Applied Biosystems) with the SYBR green method (Applied Biosystems, Foster City, CA, United States). GAPDH, β-actin and U6 were used as internal reference genes, and 2^−ΔΔCt^ method was utilized to analyze the results.

**TABLE 2:** Sequences of the primers used for qRT-PCR analysis.

**Name**	**Direction**	**Sequence (5′-3′)**
hsa_circ_0026782	Forward	CCTATAATTGGAAGGACCTGTGC
	Reverse	CCCACCATCCAACTCATCCC
hsa-miR-874-3p	Forward	CGGGCCTGCCCTGGCCCGAG
	Reverse	CAGCCACAAAAGAGCACAAT
ASB2	Forward	ACATCACGTGAGGGCCAAA
	Reverse	GCACTGAGGAAGCTGCAATC
β-actin	Forward	AGCGAGCATCCCCCAAAGTT
	Reverse	GGGCACGAAGGCTCATCATT
U6	Forward	CTCGCTTCGGCAGCACA
	Reverse	AACGCTTCACGAATTTGCGT
GAPDH	Forward	GAGCCACATCGCTCAGACAC
	Reverse	CATGTAGTTGAGGTCAATGAAGG

### Dual-Luciferase Reporter Assays

24 h before transfection, 293T cells were seeded in 48-well culture plates. The cell with 70–80% cell density was used to be transfected, and DMEM + 10% FBS was the medium. Subsequently, we diluted 1 μL of transfection reagents and plasmids (total plasmid 0.2 μg) into 25 μL of the medium, mix and incubate at room temperature for 20 min. The culture medium was transferred into the dish, and 50 μL of the transfected compound was seeded into the 48-well culture plates, followed by incubation at 37°C for 5 h. After the liquid was removed, 0.2 mL of culture medium was added and incubated at 37°C for 48 h. Then, we added 200 μl of cell lysate to each well, incubate them for 10 minutes at room temperature, and collect by centrifugation (10000 × *g*, 5 min). 20 μL of supernatant was taken and added to a 96-well luminescent plate, and then 100 μL of firefly luciferase detection solution were added. The luminescence value of the luciferase was mearsured after mixing, and 100 μL of sea kidney luciferase detection solution was added. Similarly, we redetected luminescence after mixing.

## Results

### Identification of Differentially Expressed miRNAs, circRNAs and mRNAs

**FIGURE 2:**
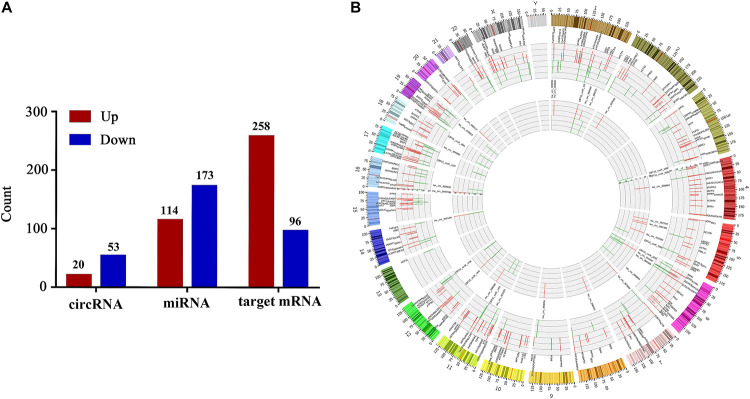
Differentially expressed circRNAs, miRNAs and target mRNAs between LD group and normal control group. Part **(A)** shows the number of DE-circRNAs, DE-miRNAs and target DE-mRNAs. Part **(B)** demonstrates the distribution of DE-circRNAs and target DE-mRNAs in the genomic level, and the up-regulated expression is shown in red, the down-regulated expression is shown in green. The height represents the degree of differential expression. DE, differentially expressed; circRNA, circular RNA; miRNA, microRNA.

Next-generation sequencing technology was implemented to detect the expressions of miRNAs, circRNAs and mRNAs in vaginal epithelial tissues of 12 women. Then, we utilized R software with edgeR/limma packages to investigate their expression differences between the LD group and the normal control group. Simultaneously, target mRNAs were also screened by correlation analysis between circRNA and mRNA with the criteria of | PCC| > 0.95 and *P* < 0.05. With the criteria of | Log_2_ (fold change)| > 1 and *P*-value < 0.05, we confirmed that the expressions of 287 miRNAs, with 114 up-regulated and 173 down-regulated miRNAs, were significantly different between the normal control group and LD group. Furthermore, 73 DE-circRNAs and 354 target DE-mRNAs were identified. Compared with the normal control group, 20 circRNAs and 258 target mRNAs were up-regulated, whereas 53 circRNAs and 96 target mRNAs were down-regulated in the LD group (Figure 2A). Circos figure was drawn to demonstrate the distribution of DE-circRNAs and target DE-mRNAs at the genomic level (Figure 2B).

### Gene Set Enrichment Analysis of DE-mRNA

**FIGURE 3:**
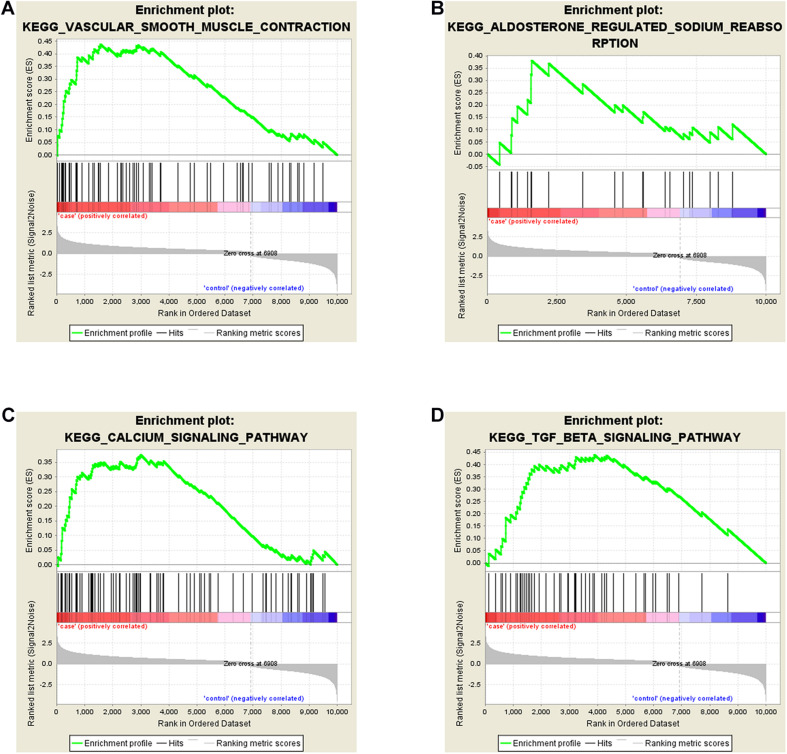
Gene Set Enrichment Analyses (GSEA) of DE-mRNAs in LD. DE-mRNAs were enriched in **(A)** Vascular Smooth Muscle Contraction, **(B)** Aldosterone Regulated Sodium Reabsorption, **(C)** Calcium Signaling Pathway, **(D)** TGF-β Signaling Pathway.

To explore the biological function of DE-mRNAs, Gene Set Enrichment Analysis was implemented, and part of the pathway results of DE-mRNAs can be seen in Figure 3. We found that DE-mRNAs were enriched in a total of 82 pathways, such as “Vascular smooth muscle contraction” (Figure 3A), “Aldosterone regulated sodium reabsorption” (Figure 3B), “Calcium signaling pathway” (Figure 3C) and “TGF-β signaling pathway” (Figure 3D), respectively. Some other pathways, like “Hypertrophic cardiomyopathy (HCM)” and “Dilated cardiomyopathy”, which are deemed to be similar to FSD in pathophysiology. Collectively, these pathways exist and are tied to the development of LD.

### Co-expression Analysis of DE-circRNAs and DE-mRNAs

73 DE-circRNAs and 354 target DE-mRNAs were selected for co-expression analysis. To investigate the correlation between them, the Pearson correlation coefficient was calculated with t.test function in the R data analysis tool. Then, 59 DE-circRNAs and 296 DE-mRNAs, which were significantly positive co-expressed, were screened with the criteria of PCC > 0.95 and *P* < 0.05 for further study.

### Construction of circRNA-miRNA Pairs

Negatively correlated pairs from Pearson and Spearman correlation analyses were determined in line with CC < −0.80 and *P* < 0.05. By merging negatively correlated pairs with interacting pairs predicted by MiRanda, 9 circRNAs and 57 miRNAs were found to construct 64 circRNA- miRNA pairs and 1 to 26 miRNAs could target one single circRNA.

### Construction of miRNA-mRNA Pairs

MiRNA can inhibit the translation of target mRNA or promote its degradation (Cloonan, 2015; Huang, 2018). So we selected negatively correlated miRNA-mRNAs pairs based on criteria of both CC < −0.80 and *P* < 0.05 and combined them with MiRanda software predicted results. Ultimately, 59 highly correlated miRNA-mRNA pairs were formed. Within the pairs, most of all were 20_5251-3p (mmu-miR-1190) which corresponded to 4 mRNAs.

### Construction of ceRNA Network

**FIGURE 4:**
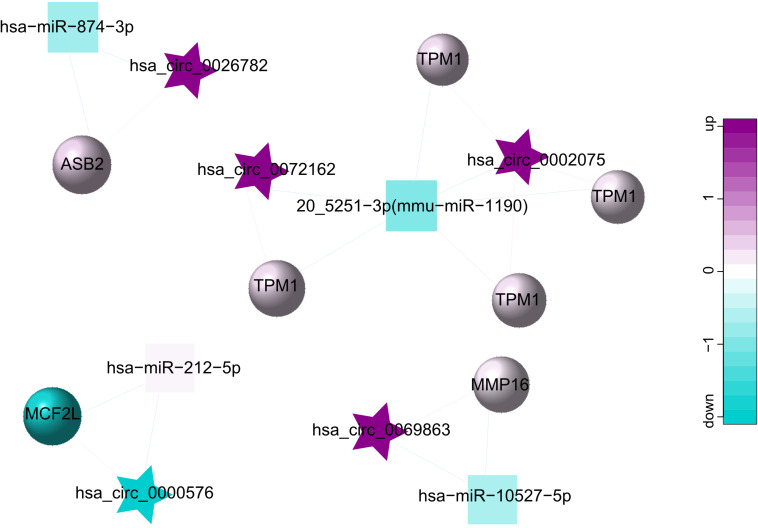
ceRNA network for 5 circRNAs (hsa_circ_0002075, hsa_circ_0000576, hsa_circ_0069863, hsa_circ_0072162, hsa_circ_0026782), 4 miRNAs (hsa-miR-212-5p, 20_5251-3p (mmu-miR-1190), hsa-miR-874-3p, hsa-miR-10527-5p) and 7 mRNAs. The star represents the circRNA, the square represents the miRNA, the circle represents the mRNA, and the edges among circRNAs, miRNAs and mRNAs represent the relationships of them. Up-regulated expression was described in purple, and down-regulated expression was described in green. The darker the color, the more significant the difference.

As a class of ceRNA, circRNA regulates mRNA by inhibiting miRNA expression. Therefore, in order to construct a ceRNA network, we integrated circRNA-miRNA pairs with miRNA-mRNA pairs. Eventually, a total of 5 circRNA nodes (hsa_circ_0002075, hsa_circ_0000576, hsa_circ_0069863, hsa_circ_0072162, hsa_circ_0026782), 4 miRNA nodes (hsa-miR-212-5p, 20_5251-3p (mmu-miR-1190), hsa-miR-874-3p, hsa-miR-10527-5p), 7 mRNA nodes and 19 edges constituted the ceRNA network, which provided a preliminary understanding of the links. Among 5 circRNAs, hsa_circ_0002075, hsa_circ_0069863, hsa_circ_0072162 and hsa_circ_0026782 were upregulated, while hsa_circ_0000576 was downregulated (Table 3). The ceRNA network showed the internal relationships between 5 circRNAs, 4 miRNAs and 7 mRNAs, which could be seen in Figure 4.

**TABLE 3:** Differentially expressed RNA in the ceRNA network.

**RNA**	**Name**	**Regulated**	**log2FC**	***P*-values**
circRNA	hsa_circ_0002075	Up	Inf	2.08E−02
	hsa_circ_0026782	Up	Inf	2.17E−02
	hsa_circ_0069863	Up	Inf	2.56E−02
	hsa_circ_0072162	Up	Inf	4.37E−02
	hsa_circ_0000576	Down	−4.04	2.90E−02
miRNA	20_5251-3p (mmu-miR-1190)	Down	−1.93	6.31E−03
	hsa-miR-874-3p	Down	−1.43	4.37E−04
	hsa-miR-10527-5p	Down	−1.28	4.27E−02
	hsa-miR-212-5p	Up	1.56	3.62E−03
mRNA	TPM1	Up	3.47	1.46E−02
	ASB2	Up	5.11	8.91E−04
	MMP16	Up	3.04	3.37E−02
	TPM1	Up	4.72	1.83E−03
	TPM1	Up	4.19	4.57E−03
	TPM1	Up	4.61	2.15E−03
	MCF2L	Down	−3.56	1.76E−02

*FC, fold change; Inf, positive infinity.*

### Verification of the Expression of RNA in ceRNA Network by RT-qPCR

**FIGURE 5:**
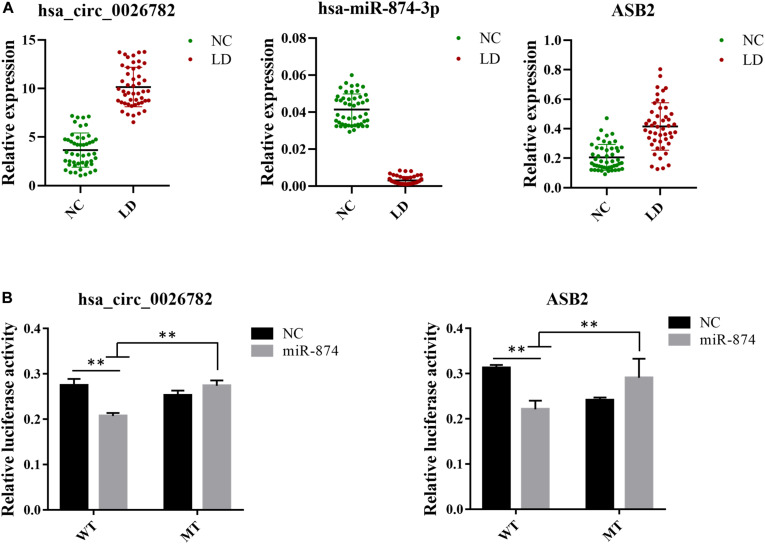
Validation of expression level and interactions among hsa_circ_0026782, hsa-miR-874-3p and *ASB2*. **(A)** Expression level of hsa_circ_0026782, hsa-miR-874-3p and *ASB2*. A total of 32 samples are used for verification. The results are the mean ± standard error of the mean (SEM) and were analyzed by *t*-tests. ^∗∗^: *P*-value < 0.01. **(B)** The direct interaction between hsa_circ_0026782, hsa-miR-874-3p and *ASB2* is confirmed by dual-luciferase reporter gene assay.

To confirm the reliability of our ceRNA network, we chosen hsa_circ_0026782, hsa-miR-874-3p and *ASB2* for validation using RT-qPCR. Results were demonstrated in Figure 5A and were consistent with that of sequencing, thus authenticating the ceRNA network was reliable. Comparing to the normal control group, hsa_circ_0026782 was significantly overexpressed in the LD group (*P* < 0.01). And the expression level of *ASB2* also increased with statistical significance in the LD group (*P* < 0.01), while hsa-miR-874-3p was downregulated (*P* < 0.01).

### Dual-Luciferase Reporter Gene Assay

To further confirm the accuracy of the ceRNA network, hsa_circ_0026782 together with its target hsa-miR-874-3p and *ASB2* were selected to validate their interaction. Luciferase reporters containing either wild-type and mutated putative binding sites of hsa_circ_0026782 were generated. Figure 5B showed that the luciferase activities of hsa_circ_0026782 wild-type (WT) reporter were significantly decreased when transfected with miR-874 mimics compared to control or hsa_circ_0026782 mutated (MUT) luciferase reporter. And a dramatic reduction in luciferase activity was also observed upon transient cotransfection of cells with the *ASB2* WT reporter and miR-874 mimics (Figure 5B). All above demonstrated direct binding between hsa_circ_0026782, miR-874 and *ASB2*.

## Discussion

In the past few years, more and more attention has been attached to FSD, and the research on FSD has developed rapidly, particularly with regard to the exploration of LD. However, the specific pathogenesis of LD is still unclear. Recently, more data has indicated that non-coding RNA may exert an important role in disease progression, particularly circRNA and miRNA (Beermann et al., 2016; Rupaimoole and Slack, 2017; Kristensen et al., 2019). CircRNA is a newly identified endogenous non-coding RNA (Meng et al., 2017). Multiple studies have ascertained that circRNA, as a class of ceRNA, plays a crucial role in certain complex diseases (Meng et al., 2017; Akhter, 2018; Aufiero et al., 2019), and differentially expressed circRNAs have also been observed in the vaginal epithelium of women with LD (Zhang J. et al., 2019). In addition, our previous study has revealed that overexpressed miR-137 is involved in LD by down-regulating *AQP2* (Zhang H. et al., 2018). Nevertheless, the interactions between circRNAs and miRNAs in LD are mostly unknown. Therefore, we selected women’s vaginal epithelial tissues for analysis and then identified differentially expressed miRNAs, circRNAs and mRNAs between the LD group and normal control group. Hereafter, we explored the potential pathways of DE-mRNAs in LD by using GESA. The relationships between miRNAs, circRNAs and mRNAs in LD were explored and exhibited in the ceRNA network. And we also performed RT-qPCR and dual-luciferase reporter gene assay to verified the expression level and interactions of genes in the network. These results provide a new perspective for understanding the occurrence of LD.

In the current study, through next-generation sequencing technology, we successfully identified dysregulated miRNAs, circRNAs and mRNAs in the LD group compared with the normal control group. Total 73 DE-circRNAs, 287 DE-miRNAs and 354 target DE-mRNAs were selected with the criteria as |Log2 (fold change)| > 1 and *P*-value < 0.05 for further analysis, and the research on them is meaningful.

For understanding the potential pathways and biological functions of the significantly deregulated mRNAs in the occurrence and development of LD, GESA was performed. The results revealed that DE-mRNAs were primarily enriched in “Vascular smooth muscle contraction,” “Aldosterone regulated sodium reabsorption,” “Calcium signaling pathway” and “TGF-β signaling pathway,” which may be closely related to LD. In the past research, vascular smooth muscle contraction had been shown to reduce blood flow to the genitals, leading to insufficient vaginal lubrication and weak clitoral erections (Marthol and Hilz, 2004; Comeglio et al., 2016). Meanwhile, the calcium signaling pathway may also affect LD because the increase in intracellular Ca^2+^ can stimulate numerous downstream proteins and cause the contractile response of vascular smooth muscle (Morgado et al., 2012; Kuo and Ehrlich, 2015). As noted above, these results showed that DE-mRNAs was involved in many LD-associated biological metabolic pathways, and changes in vaginal blood flow may be an important cause of LD. Furthermore, DE-mRNAs were also enriched in “Aldosterone regulated sodium reabsorption” and “TGF-β signaling pathway,” which were involved in affecting fluid transport and vaginal tissue fibrosis (Park et al., 2001). It is generally known that vaginal lubrication is mediated by fluid transport, which is closely related to blood flow and vaginal epithelial ion transport (Pei et al., 2013; Sun et al., 2016). Moreover, Park et al. (2001) also indicated that vaginal tissue fibrosis may result in the reduction of vaginal lubrication in diabetic women. All the information above provided some insights into the pathogenesis of LD.

As a miRNA sponge, circRNA can function as ceRNA and competitively bind to miRNAs, which in turn affects mRNA expression (Han et al., 2018; Li X. et al., 2018; Kristensen et al., 2019). In this current study, we have found that 5 circRNAs could regulate the expressions of 7 genes by conjugating with hsa-miR-212-5p, 20_5251-3p (mmu-miR-1190), hsa-miR-874-3p or hsa-miR-10527-5p. These results provide evidence that 5 circRNAs can participate in the occurrence of LD through ceRNA regulatory mechanisms. Of them, through dual-luciferase reporter assay, hsa_circ_0026782 has been verified that it directly targets miR-874. In accordance with these results, Shen et al. (2020) also reported that miR-874-3p was the predicted MREs for hsa_circ_0026782, which confirms the accuracy of our results. In addition, several miRNAs in ceRNA network were reported previously to be involved in the cell cycle, angiogenesis, mucosal fibrosis, and so on. K. Xie et al. (2020) detected that miR-874-3p can inhibit the C-X-C motif chemokine ligand 12 (CXCL12) expression, thus promote angiogenesis in ischemic stroke. Kumarswamy et al. (2014) argued that miR-212 accompanied by strong endothelial inhibitory effects, and adversely affected angiogenesis. In addition, the role of miR-874 in facilitating proliferation and apoptosis in many diseases has also been confirmed (Wang et al., 2014; Witek et al., 2020; Zhang et al., 2020). And miR-212 was demonstrated that can target SMAD7, thereby facilitating the activation of TGF-β (a master fibrogenic cytokine) and downstream pathway *in vitro* experiments (Zhu et al., 2018). As we all know, LD involves processes such as vaginal fibrosis and blood vessel changes (Park et al., 1997; Goldstein and Berman, 1998; Castiglione et al., 2015), suggesting that these miRNAs may play regulatory roles in LD.

However, there are still several limitations to this study. First, our experiment also needs to expand the sample size for research. Secondly, we explored some mechanisms of LD, discovered the interactions among circRNAs, miRNAs and mRNAs in LD, but this only depended on biological information technology. It still needs further study with the help of cell and animal models, and we will continue to do so in the future.

## Conclusion

In summary, we identified differentially expressed genes in the vaginal epithelium of women with LD, and constructed a ceRNA network based on the hypothesis. Then, the results of RT-qPCR and dual-luciferase reporter gene assay confirmed that interactions among circRNA, miRNA and mRNA were involved in LD. However, we didn’t identify it in cell and animal models, so more works were necessary to explore potential molecular markers for the occurrence of LD.

## Data Availability Statement

The datasets can be acccessed here: https://www.ncbi.nlm.nih.gov/sra/PRJNA646352.

## Ethics Statement

The studies involving human participants were reviewed and approved by Ethics Review Committee of Nanjing Maternity and Child Health Care Hospital. The patients/participants provided their written informed consent to participate in this study.

## Author Contributions

LP, AZ, and JM conceived and designed the experiments. SC, AZ, JL, and JF collected the data. JM, LP, JZ, SC, and JL involved in data analysis and interpretation. SC and JL drafted the article. SC, JZ, JF, JM, AZ, and LP revised it for intellectual content. SC was responsible for data curation. All authors contributed to the article and approved the submitted version.

## Conflict of Interest

The authors declare that the research was conducted in the absence of any commercial or financial relationships that could be construed as a potential conflict of interest.

## Footnotes

1 http://www.broadinstitute.org/gsea
